# A_2A_ Adenosine Receptors Are Differentially Modulated by Pharmacological Treatments in Rheumatoid Arthritis Patients and Their Stimulation Ameliorates Adjuvant-Induced Arthritis in Rats

**DOI:** 10.1371/journal.pone.0054195

**Published:** 2013-01-11

**Authors:** Fabrizio Vincenzi, Melissa Padovan, Martina Targa, Carmen Corciulo, Sarah Giacuzzo, Stefania Merighi, Stefania Gessi, Marcello Govoni, Pier Andrea Borea, Katia Varani

**Affiliations:** 1 Department of Clinical and Experimental Medicine, Pharmacology Section, University of Ferrara, Ferrara, Italy; 2 Department of Clinical and Experimental Medicine, Rheumatology Section, University of Ferrara, Ferrara, Italy; Virgen Macarena University Hospital, School of Medicine, Spain

## Abstract

A_2A_ adenosine receptors (ARs) play a key role in the inhibition of the inflammatory process. The purpose of this study was to evaluate the modulation of A_2A_ARs in rheumatoid arthritis (RA) patients after different pharmacological treatments and to investigate the effect of A_2A_AR stimulation in a rat model of arthritis. We investigated A_2A_AR density and functionality in RA progression by using a longitudinal study in RA patients before and after methotrexate (MTX), anti-TNFα agents or rituximab treatments. A_2A_ARs were analyzed by saturation binding assays in lymphocytes from RA patients throughout the 24-month study timeframe. In an adjuvant-induced arthritis model in rats we showed the efficacy of the A_2A_AR agonist, CGS 21680 in comparison with standard therapies by means of paw volume assessment, radiographic and ultrasonographic imaging. Arthritic-associated pain was investigated in mechanical allodynia and thermal hyperalgesia tests. IL-10 release following A_2A_AR stimulation in lymphocytes from RA patients and in serum from arthritic rats was measured. In lymphocytes obtained from RA patients, the A_2A_AR up-regulation was gradually reduced in function of the treatment time and the stimulation of these receptors mediated a significant increase of IL-10 production. In the same cells, CGS 21680 did not affected cell viability and did not produced cytotoxic effects. The A_2A_AR agonist CGS 21680 was highly effective, as suggested by the marked reduction of clinical signs, in rat adjuvant-induced arthritis and associated pain. This study highlighted that A_2A_AR agonists represent a physiological-like therapeutic alternative for RA treatment as suggested by the anti-inflammatory role of A_2A_ARs in lymphocytes from RA patients. The effectiveness of A_2A_AR stimulation in a rat model of arthritis supported the role of A_2A_AR agonists as potential pharmacological treatment for RA.

## Introduction

Rheumatoid Arthritis (RA) is a chronic autoimmune disease that primarily affects joints and causes pain, stiffness, swelling and limited motion [1], [2]. In RA the inflammatory process leads to progressive cartilage degradation with synovial hyperplasia, change in underlying bone with erosions and high levels of pro-inflammatory mediators [3], [4]. It is widely accepted that cytokines such as tumor necrosis factor α (TNFα) and interleukin (IL) family mediate a large variety of effector functions in the context of RA pathogenesis [5]. Conversely, the anti-inflammatory cytokine IL-10 is relatively unique in its ability to down-regulate the production of multiple pro-inflammatory cytokines, leading to the notion that IL-10 may modulate the disease expression in RA [6]. Early diagnosis and therapy are crucial in order to prevent unfavourable outcome avoiding joint deterioration and functional disability [7]. Conventional disease modifying anti-rheumatic drugs (DMARDs) such as methotrexate (MTX) in monotherapy or in combination are currently the first medications usually prescribed in RA patients [8], [9]. More recently, the introduction of anti-TNFα agents (e.g. adalimumab, etanercept) or the use of anti-CD20 B cell targeted therapy (rituximab, RTX) have provided a marked improvement in RA even if some patients do not respond or fail to maintain adequate response to these treatments [10], [11]. Therefore the availability of alternative therapeutic options, targeting the pathways associated to inflammatory processes in RA, could be very useful to assist clinicians in making alternative pharmacological treatment choices [12], [13].

Adenosine, a purine nucleoside, regulates the mechanisms of inflammation acting with four cell surface receptors named as A_1_, A_2A_, A_2B_ and A_3_ARs which are coupled to different G proteins [14], [15]. In particular, A_2A_ARs are targeted to be anti-inflammatory receptors and their activation suppress the elevated levels of pro-inflammatory cytokines [16]. It has been shown that A_2A_AR agonists are able to increase the production of the anti-inflammatory cytokine IL-10 in various *in vitro* and *in vivo* models [17], [18]. In human synoviocytes A_2A_AR stimulation decreased TNFα, IL-6 and IL-8 production and increased IL-10 release showing a marked down-regulation of the inflammatory status [19], [20].

The anti-inflammatory and tissue-protective effects elicited by A_2A_ARs have been reported in several *in vivo* models of inflammatory diseases [21]. At present, different animal models of arthritis have been used to test novel therapeutic hypothesis, their efficacy and the presence of adverse effects [22], [23]. It has been shown that A_2A_AR stimulation ameliorated clinical signs and improved histological damage in murine collagen-induced arthritis model [24]. The treatment with A_2A_AR agonists decreased the expression of inflammatory cytokines and the degree of oxidative and nitrosative damage [25]. Adjuvant-induced arthritis is a commonly used model of inflammatory arthritis, with an incidence of around 90%, making it an ideal model in which to investigate arthritic changes and to evaluate compounds that might be of potential use as drugs for RA treatment [26], [27]. Recently, A_2A_AR agonists have been approved for clinical trials based on their anti-inflammatory and wound-healing properties [15], [28], [29]. In clinical evaluation, the chronic A_2A_AR activation was able to reduce the inflammatory status in specific diseases as diabetic nephropathy and to promote healing of diabetic foot ulcers [30].

In a previous study our group showed that A_2A_ and A_3_ARs are up-regulated in early RA patients and after MTX treatment but not in RA patients treated with anti-TNFα agents [31]. An inverse correlation between A_2A_ and A_3_AR density with disease activity score (DAS) was found in RA patients. Moreover, A_2A_ and A_3_AR stimulation mediated a reduction of pro-inflammatory cytokines and matrix metalloproteinases production suggesting a role of adenosine in the inhibition of inflammation and cartilage degradation [32].

The aim of the present study was to evaluate in RA patients the influence of different biologic therapies as anti-TNFα drugs or RTX in comparison with MTX treatment on A_2A_AR density at various time points of treatment (from 0 to 24 months). In RA patients treated with RTX the relationship between A_2A_AR density and DAS 28 values was investigated. The changes of A_2A_AR expression after different pharmacological treatments in function of the time and the agonist-induced IL-10 production have suggested the potential use of A_2A_AR stimulation as RA therapeutic approach. As a consequence, in a complete Freund’s adjuvant (CFA)-induced arthritic rat model, the effect of a well known A_2A_AR agonist named 2-[p-[2-carboxyethyl)-phenetyl-amino]-5′-N-ethyl-carboxamido-adenosine (CGS 21680) was evaluated in controlling rat adjuvant arthritis and associated pain.

## Materials and Methods

### Ethics Statement

The study was approved by the local Ethic Committee of the University Hospital of Ferrara (Italy) and written informed consent was obtained from each participant in accordance with the principles outlined in the Declaration of Helsinki.

All animal procedures were conformed to the Italian guidelines for the use of animals in biomedical research (authorization from the Italian Ministry for Health 122/2011-B).

### Sample Collection and Human Lymphocyte Preparation

Lymphocytes were isolated and prepared as previously described from the peripheral blood of control subjects and RA patients [31], [32]. The isolation of blood cells started no later than 3 to 4 hours after the samples had been taken. The blood was supplemented with 6% (by weight) Dextran T500 solution (Sigma-Aldrich, St Louis, MO, USA) and erythrocytes were allowed to settle down for 60 min. Leukocytes were pelleted by centrifugation for 15 min at 100×g and the remaining erythrocytes were lyzed in distilled water at 4°C rapidly restoring the isotonicity by NaCl solution. Then, the cells were pelleted by centrifugation for 5 min at 250×g, suspended in Krebs-Ringer phosphate buffer and layered onto 10 ml of Fycoll-Hypaque (GE Healthcare, Little Chalfont, UK). After centrifugation, mononuclear cells were washed in 0.02 M phosphate-buffered saline (PBS) at pH 7.2 containing 5 mM MgCl_2_ and 0.15 mM CaCl_2_. Finally, they were decanted into a culture flask and placed in a humidified incubator (5% CO_2_) for 2 hours at 37°C. This procedure, aimed at removing monocytes which adhere to the culture flasks, resulted in a purified lymphocyte preparation containing at least 99% small lymphocytes identified by morphological criteria. To obtain membrane suspensions, cell fractions were centrifuged in hypothonic buffer at 20000×g for 10 min. The resulting pellet was resuspended in tris HCl 50 mM buffer pH 7.4 containing 2 UI/ml adenosine deaminase (Sigma-Aldrich) and incubated for 30 min at 37°C. After the incubation the suspension was centrifuged again at 40000×g for 10 min and the final pellet was used for radioligand binding assays. The protein concentration was determined by a Bio-Rad method with bovine albumine as reference standard [19].

### Saturation Binding Experiments to A_2A_ARs

Saturation binding to A_2A_ARs was carried out with the use of [^3^H]-4-(2-[7-amino-2-(2-furyl) [1], [2], [4]-triazolo[2,3-a] [1], [3], [5] triazin-5-ylamino] ethyl) phenol ([^3^H]-ZM 241385, specific activity 27 Ci/mmol, Biotrend, Cologne, Germany), as radioligand [31], [32]. Cell membranes (60 µg of protein) were incubated for 60 min at 4°C with various concentrations (0.1–20 nM) of [^3^H]-ZM 241385. Non specific binding was determined in the presence of 5-amino-7-(phenylethyl)-2-(2-furyl)-pyrazolo[4,3-e]-1,2,4-triazolo[1,5-c]pyrimidine (SCH 58261, 1 µM, Tocris, Bristol, UK) and was always <25% of the total binding. Bound and free radioactivity were separated by filtering the assay mixture through Whatman GF/B glass fiber filters by using a Brandel cell harvester. The filter bound radioactivity was counted in a 2810 TR liquid scintillation counter (Perkin-Elmer, Boston, MA, USA).

### IL-10 Release in Cultured Lymphocytes

Isolated lymphocytes from untreated RA patients were suspended at a density of 10^6^ cells/ml in RPMI 1640 medium supplemented with 2% fetal bovine serum (Euroclone, Milan, Italy) and seeded into 24-well plates. Lymphocytes were incubated for 24 hours in the absence or in the presence of CGS 21680 (1 nM–10 µM). A selective A_2A_AR antagonist SCH 58261 (1 µM) was also used to verify the specific involvement of these receptors in IL-10 release. At the end of incubation, the cell suspension was collected and centrifuged at 1000×g for 10 min at 4°C. The anti-inflammatory cytokine IL-10 levels were determined with a quantitative sandwich ELISA kit (R&D Systems, Minneapolis, MN, USA) according to the manufacturer instructions [33]. The reaction was developed with streptavidin-horseradish peroxidase and optical density was read at 450 nm wavelength.

### Cell Viability and Cytotoxicity Assays

MTT and lactate dehydrogenase (LDH) assays were performed in lymphocytes from untreated RA patients after 24 hours of incubation with different concentration of CGS 21680 (1 nM–10 µM). In the MTT assay, the yellow tetrazolium salt is reduced in metabolically active cells to form insoluble purple formazan crystals, which are solubilized by the addition of a detergent. Briefly, 100 µl of cell suspension was placed in one well of a 96-well tissue culture plate, and 10 µl of MTT solution (2.5 mg/ml; Sigma-Aldrich) was added. After incubation for 4 hours at 37°C, 100 µl of acid-isopropanol (0.04 N HCl in isopropanol) was added and mixed by gentle pipetting to solubilize the cells. The optical density of the solution was read at 550 nm using a microplate reader [34].

LDH activity can be used as an indicator of cell membrane integrity and serves as general meaning to assess cytotoxicity resulting from chemical compounds or environmental toxic factors. LDH assay was performed in human lymphocytes from untreated RA patients as previously described [35]. At the end of incubation with CGS 21680 for 24 hours, 100 µl of supernatant per well was harvested and transferred into a new 96-well, flat-bottom plate. LDH substrate (100 µl) was added to each well and incubated for 30 min at room temperature protected from light. The absorbance of the samples was measured at 490 mm on spectrophotomer.

### Animals and Treatments

Male Sprague-Dawley rats with a mean weight of 150−180 g were obtained from Charles River Laboratories (Milan, Italy) and maintained in a climate-controlled environment under 12 hours light/12 hours dark conditions. The animals were fed standard rodent chow and water ad libitum. Adjuvant-induced arthritis was elicited in rats according to established methods [36]. Briefly, rats were injected subcutaneously in the plantar surface of the left hind paw with 100 µl of CFA (1 mg/ml) containing heat killed Mycobacterium Tubercolosis suspended in paraffin oil (Sigma-Aldrich). The rats (n = 30) were subsequently divided into five groups of six rats each. A stock solution of CGS 21680 were prepared in DMSO and further diluted in PBS for an injection volume of 0.3 ml per rat. Group 1 was injected with CFA but not pharmacologically treated. Group 2 represented the sham group where the rats were injected with PBS solution. Group 3 was treated with MTX (2 mg/kg) administered subcutaneously (s.c.) in a single dose a week. Group 4 was treated with CGS 21680 at the dose of 0.1 mg/kg intraperitoneally (i.p.) three times a week. Group 5 was treated with etanercept at a single dose of 5 mg/kg a week, s.c. The pharmacological treatments started 1 week after CFA injection. Clinical evaluation of arthritis was performed throughout the study and animals were sacrificed on day 28.

### Mechanical Allodynia Evaluation

Paw withdrawal thresholds were determined using the Dynamic Plantar Aesthesiometer (Ugo Basile, Milan, Italy), an apparatus that generates a mechanical force linearly increasing with time [37]. Rats were placed individually in plastic cages with a wire mesh bottom and allowed to acclimatize for at least 2 hours. Increasing mechanical stimulation (2.5 g/s, cut-off force: 50 g) was applied to the plantar surface of a hind paw. The nociceptive threshold was defined as the force, in grams, at which the rat withdraw its paw. When a withdrawal response occurred, the stimulus was terminated and the response threshold electronically measured.

### Measurement of Thermal Hyperalgesia

The response to noxious thermal stimulus was determined using a Plantar Test Apparatus (Ugo Basile) [38]. Briefly, animals were placed into a Plexiglas chamber for 2 hours acclimatization period followed by testing. A movable infrared radiant heat source was placed directly under the plantar surface of the hind paw and the time taken for hind paw withdrawal was monitored (withdrawal latency). A cut-off latency of 30 s was imposed to avoid tissue damage and the mean of three values was used for data analysis.

### Assessment of Paw Volume

The rat pawvolume was measured before CFA injection and at day 1, 7, 14, 21, and 28 after CFA injection using a plethysmometer (Ugo Basile) [39]. The plethysmometer consists of 2 vertical interconnected water-filled Perspex cells, the larger of which is used to measure volume displacement produced by dipping the rat paw in.

### Radiographic Analysis

On day 28, rats were anesthetized and placed on Fuji Medical X-ray and exposed to an X-ray source for 125 ms at 50 kV_p_, 125 mA (15.6 mA/s). The X-ray film was developed in a one in five dilution of Agfa G150 Developer for 2.5 min and fixed using a one in four dilution of Agfa G354 Fixing bath for 2.5 min. Radiographs were placed in slide mounts and projected [36]. The radiological alterations were arbitrarily graded between 0 and 3 according to the severity of the swelling of the soft tissue around the joints of the hind paws, osteoporosis, periarticular bone erosion and narrowing of the joint space. The radiographic score was: grade 0 =  normal; grade 1 =  mild change compared to normal; grade 2 =  moderate change compared to normal; grade 3 =  marked change compared to normal.

### Ultrasonographic Assessment

On days 7, 21 and 28 joint ultrasound (US) assessment was performed by using an Aplio XG (Toshiba, Tokyo, Japan) scanner with 12 MHz linear transducer. The US assessment included transverse and longitudinal scanning of left paw, lateral dorsal view. Synovial power Doppler (PD) was evaluated selecting a region that included bony margins, joint space and a variable view of surrounding tissues such as ankle joint. Moreover, joint effusion, synovitis and bone erosions was assessed by grey-scale scan and PD activity. Pulse repetition frequency was adjusted at the lowest permissible to maximize sensitivity. Color gain was set just below the level that causes the appearance of noise artefacts. A total US score was assigned semiquantitatively (grade 0–3) by a combination of grey-scale scan and PD signals [40], [41]. In particular, effusion/synovitis was graded as: 0 =  absent; 1 =  mild; 2 =  moderate; 3 =  marked. Bone erosion was graded as: 0 =  smooth bone surface; 1 =  irregularities of the bone surface, in the absence of discontinuities displayable on two scanning planes; 2 =  discontinuity shown by two scanning planes; 3 =  marked bone destruction. Power Doppler was graded as: 0 =  normal, absence of flow signals; 1 =  mild, isolated flow signals; 2 =  moderate, vases confluent; 3 =  marked, multiple signals over half of the intra-articular surface.

### Western Blotting Analysis and IL-10 Levels

Rats were killed by decapitation 28 days after CFA injection, the spleen was removed and a cell suspension of splenocytes was prepared by using sterilized nylon mesh gauze in the presence of sterile saline. Mononuclear cells were isolated by centrifugation for 15 min at 500×g of the cell suspension through Fycoll-Hypaque [42]. Purified lymphocyte preparation was primarily obtained as described above for human lymphocytes. The cells were lysed in Triton lysis buffer [32] and aliquots of total protein samples (50 µg) were analysed using a specific rat A_2A_AR antibody (Alpha Diagnostic, San Antonio, TX, USA). Filters were washed and incubated for 1 hour at room temperature with peroxidase-conjugated secondary antibody (1∶2000 dilution). Specific reaction were revealed with enhanced chemiluminescence Western blotting detection reagent (GE Healthcare). Western blotting assays were also normalized against the housekeeping protein GAPDH.

Rat blood were collected, centrifuged at 3000×g for 10 min, and serumwas separated and stored at −20°C until assay.IL-10 levels in rat serum samples were evaluated by using an ELISA kit following manufacturer’s instruction (R&D System).

### Data and Statistical Analysis

Dissociation equilibrium constants for saturation binding, affinity or K_D_ values, as well as the maximum densities of specific binding sites, Bmax were calculated for a system of one or two-binding site populations by non-linear curve fitting using the program Ligand purchased from Kell Biosoft [19]. All data are reported as mean ± SD except for radiographic and ultrasonographic scores that are expressed as median and interquartile range. Statistical analysis of the data was performed by repeated measures analysis of variance (ANOVA) followed by Dunnett’s t-test or unpaired two-sided Student’s t-test for comparison of two samples. In the case of radiological or US scoring, Wilcoxon’s nonparametric analysis of variance for comparison of two samples was performed. All analysis were carried out using GraphPad Prism 5.0 statistical software package and differences were considered statistically significant with a *p* value less than 0.05 (Graph Pad Software, San Diego, CA, USA).

## Results

### Patients and Control Subjects

All patients enrolled in this study were recruited from the Rheumatology Section, Department of Clinical and Experimental Medicine, University of Ferrara, Italy. A total of 60 patients were included and divided in RA patients treated with MTX (n = 24), with anti TNFα agents (adalimumab or etanercept, n = 12) or with RTX (n = 24). RA patients with established disease fulfilled the American College of Rheumatology (ACR) 1987 criteria for RA [43]. The demographic, clinical and pharmacological details are listed in Table 1. All patients have continued to be regularly monitored during the timeframe of the study. Clinical information about disease characteristics such as disease onset, previous and current therapy, rheumatoid factor (RF) and anti-cyclic citrullinated peptide antibodies (ACPA) presence were reported (Table 1). Disease Activity Score evaluated in 28 joints (DAS 28) and Health Assessment Questionnaire (HAQ) were completed for all patients [44], [45]. The statistical analysis of the clinic parameters such as DAS 28 and HAQ reveals a significant difference between the groups that is considered quite common within a cohort of RA patients. Healthy controls (n = 60), matched for similar age to RA patients, were volunteers from Ferrara University Hospital Blood Bank.

**Table 1:** Clinical features and pharmacological treatments in RA patients.

	Control Subjects (n = 60)	RA patients MTXtreated (n = 24)	RA patients RTX treated (n = 24)	RA patients Anti-TNFα treated (n = 12)
**Clinical Parameters**				
N° female/male	45/15	20/4	22/2	8/4
Age, mean ± SD years	45.3±36.4	48.2±19.1	46.0±9.8	41.0±13.9
Disease Duration, mean ± SD months	–	67.5±69.1	127.7±81.3**	120.0±100.1
Previous DMARDs, no(range)	–	2 (1–4)	2 (1–5)	2 (1–3)
No. (%) RF+	–	15 (63)	20 (83)	7 (58)
No. (%) ACPA+	–	12 (50)	19 (79)	7 (58)
Baseline DAS 28, mean± SD	–	4.93±0.88	5.57±1.18*	4.72±0.80#
Baseline HAQ score, mean ± SD	–	1.32±0.59	1.70±0.78	1.13±0.52#
2 yrs delta DAS 28 mean ± SD		−1.01±1.18	−2.31±2.84*	−2.07±0.10**
**Pharmacologic treatments, no. (%)**				
NSAIDs		2(8)	–	–
Low-dose steroids (4–5 mg/day)		20 (83)	20 (83)	8 (67)
Steroids+DMARDs		24 (100)	11 (46)	5 (42)
Methotrexate (10–20 mg/week)		24 (100)	9 (37)	7 (58)
Hydroxychloroquine (200 mg/day)		18 (75)	–	2 (17)
Azatioprine (50 mg/day)		–	1 (4)	–
Leflunomide (20mg/day)		–	1 (4)	1 (8)
Etanercept(50 mg/week)		–	–	5 (42)
Adalimumab (40 mg/week)		–	–	7 (58)
Rituximab (2x1000mg/24 weeks)		–	24 (100)	–

RA = rheumatoidarthritis; RF =  rheumatoidfactor; DMARDs = disease-modifyingantirheumaticdrugs; ACPA = anti-cycliccitrullinatedpeptideantibodies; DAS = diseaseactivity score; HAQ = healthyassessmentquestionnaire; NSAIDs = nonsteroidal anti-inflammatorydrugs, MTX = methotrexate; RTX = rituximab.

* p<0.05 vs MTX-treated RA patients;

** p<0.01 vs MTX-treated RA patients;

# p<0.05 vs RTX-treated patients.

### Binding Parameters of A_2A_ARs in Lymphocytes of RA Patients

**Figure 1:**
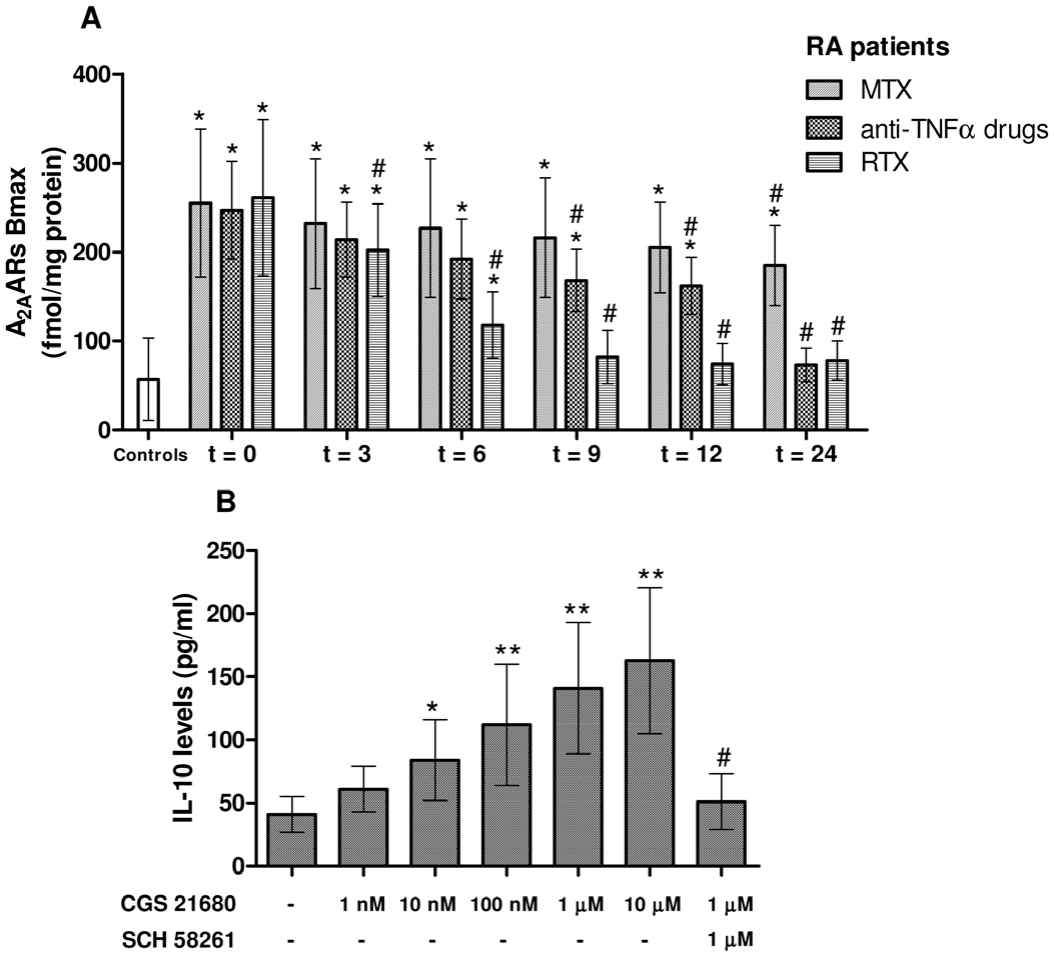
A_2A_AR upregulation in RA patients and *in vitro* stimulation by CGS 21680 increased IL-10 production. (A) A_2A_AR Bmax values in lymphocytes from RA patients evaluated at different time points of treatment with MTX, anti-TNFα drugs or RTX. Data are expressed as mean ± SD. *, *p<*0.01 vs healthy controls (white bar, Bmax = 57±46 fmol/mg protein); #, *p*<0.01 vs t = 0. (B) Effect of CGS 21680 (1 nM–10 µM) and SCH 58261 (1 µM) on IL-10 production in cultured lymphocytes from untreated RA patients. Data are expressed as mean ± SD. *, *p*<0.05vs basal; **, *p*<0.01 vs basal; #, *p*<0.01 vs CGS 21680 (1 µM).

Affinity (K_D_) and density (Bmax) of A_2A_ARs were evaluated in lymphocyte membranes obtained from RA patients at different time points of treatment with MTX, anti-TNFα agents or RTX (Table 2). Before the treatment start, RA patients revealed an up-regulation of A_2A_AR density and a lower affinity if compared with age matched healthy controls (K_D_ = 1.34±0.85 nM; Bmax = 57±46 fmol/mg protein). In MTX-treated patients, A_2A_AR affinity and density were found to be significantly different from control subjects in the time points investigated, even if a significant reduction of K_D_ and Bmax values after 24 months of treatment respect to the beginning of the study was observed. In lymphocytes from RA patients treated with anti-TNFα drugs, a statistically significant decrease of A_2A_AR density was obtained after 9 months of treatment. In the same patients the A_2A_AR Bmax values were similar to control subjects after 24 months from the beginning of the treatment. Interestingly, RTX-treated patients showed a significant reduction of Bmax and K_D_ after 3 months of treatments reaching values similar to control subjects at the subsequent time points investigated (from 6 to 24 months). Fig. 1A summarises the A_2A_AR density values obtained in lymphocytes from RA patients treated with MTX, anti-TNFα agents or RTX after 0, 3, 6, 9, 12 and 24 months from the treatment start.

**Table 2:** A_2A_AR affinity (K_D_, nM) and density (Bmax, fmol/mg protein) in lymphocytes from RA patients at different time points of treatment.

A_2A_ARs K_D_, nM Bmax, fmol/mg protein	RA patients MTX treated	RA patients anti-TNFα treated	RA patients RTX treated
**t = 0**	2.61±0.64* 255±83* n = 24	2.64±0.52* 247±55* n = 12	2.59±0.54* 261±88* n = 24
**t = 3**	2.24±0.54* 232±73* n = 24	2.16±0.45* 214±42* n = 12	2.06±0.56*^#^ 202±52*^#^ n = 22
**t = 6**	2.18±0.59* 227±78* n = 24	2.17±0.38* 192±45* n = 12	1.81±0.32*^#^ 118±37*^#^ n = 21
**t = 9**	2.14±0.58* 216±67* n = 20	2.08±0.35*^#^ 168±35*^#^ n = 10	1.25±0.34^#^ 82±30^#^ n = 18
**t = 12**	2.12±0.59* 205±51* n = 18	2.02±0.32*^#^ 162±34*^#^ n = 10	1.05±0.23^#^ 74±23^#^ n = 15
**t = 24**	1.95±0.34*^#^ 185±45*^#^ n = 14	1.27±0.38^#^ 73±19^#^ n = 10	1.26±0.32^#^ 78±22^#^ n = 10

Healthy controls (n = 60): K_D_ = 1.34±0.85 nM; Bmax = 57±46 fmol/mg protein. MTX = methotrexate; RTX = rituximab. Time (t) is expressed as months of pharmacological treatment. Data are expressed as mean ± SD. Differences were considered significant at a value of *p*<0.01 vs healthy controls (*) or vs t = 0 (#).

### Effect of A_2A_AR Stimulation on IL-10 Release from Lymphocytes of RA Patients

In cultured lymphocytes obtained from untreated RA patients (n = 30), the A_2A_AR agonist CGS 21680 was able to induce a dose-dependent significant increase of the anti-inflammatory cytokine IL-10 levels (Fig. 1B). In particular, CGS 21680 at 1 µM and at 10 µM concentration determined a marked release of IL-10 with an increase of 3.3 and 4.0 fold versus basal condition (*p*<0.01), respectively. The effect of A_2A_AR stimulation on IL-10 release was completely blocked by the A_2A_AR selective antagonist, SCH 58261(Fig. 1B). These data strongly support the potential use of A_2A_AR agonist in RA as anti-inflammatory agent and, consequently, a series of *in vivo* experiments were performed to test this hypothesis.

### CGS 21680 did not Affect Cell Viability and did not have Cytotoxic Effects on Human Lymphocytes

The cell viability was assessed in human lymphocytes obtained from untreated RA patients exposed for 24 hours to different concentrations (1 nM –10 µM) of CGS 21680. The A_2A_AR agonist did not affect cell viability at all the tested concentrations respect to the control condition. In particular, at the highest concentration tested (10 µM) CGS 21680 showed a mean absorbance value of 0.63±0.12 that was not statistically different from the mean value obtained in control condition (0.59±0.10).

The A_2A_AR agonist CGS 21680 did not modify LDH release in human lymphocytes at the different tested concentrations (1 nM –10 µM). The mean absorbance value obtained with CGS 21680 at the 10 µM concentration was 0.32±0.06 and it did not differ from control condition (0.35±0.08). These results suggested that the treatment with GCS 21680 was not cytotoxic for human lymphocytes.

### The A_2A_AR Density and DAS 28 Gradually Reduced in RA Patients Treated with RTX

**Figure 2:**
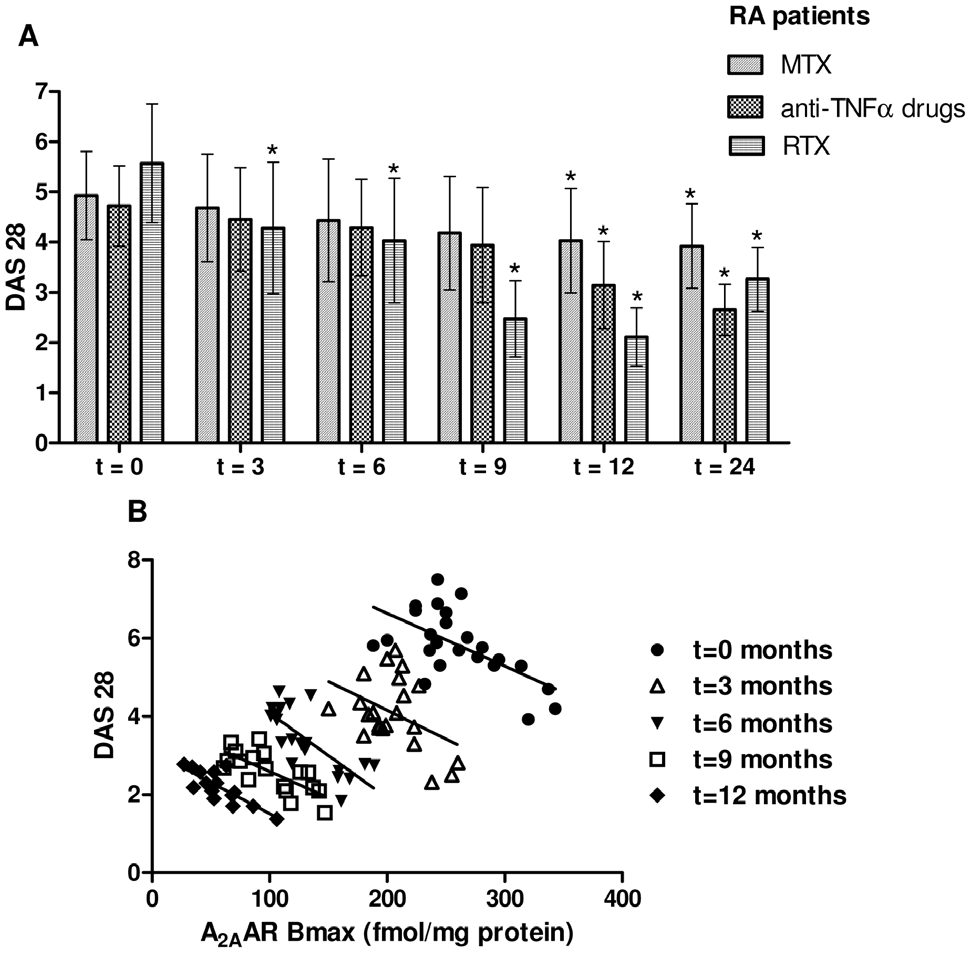
A_2A_AR Bmax and DAS 28 values gradually reduced in function of the time of treatment. (A) DAS 28 values in RA patients evaluated at different time points of treatment with MTX, anti-TNFα drugs or RTX. Data are expressed as mean ± SD.*, *p*<0.01 vs t = 0 months. (B) Time-dependent relationship between A_2A_AR Bmax and DAS 28 in lymphocytes from RTX treated RA patients.

To date the 28 joint disease activity score (DAS 28) represents a validated composite index to assess RA disease activity. Fig. 2A reports the DAS 28 values in RA patients evaluated at different time points of treatment with MTX, anti-TNFα drugs or RTX. To investigate the involvement of A_2A_ARs in RA progression we have studied the association between these receptor subtypes and DAS 28 in function of the time of treatment. Interestingly, in RTX-treated patients the DAS 28 reduction (from 5.57 to 2.11) respect to the time of treatment was closely associated to a significant A_2A_AR decrease of 3.5 fold after 12 months respect to RA untreated patients (Fig. 2B, Table 2). In particular, the time-dependent relationship (from 0 to 12 months) between A_2A_ARs and DAS 28 in lymphocytes from RA patients treated with RTX revealed a significant reduction of these parameters (Fig. 2B).

### Effect of CGS 21680 on CFA-induced Arthritis in Comparison with MTX and Etanercept

**Figure 3:**
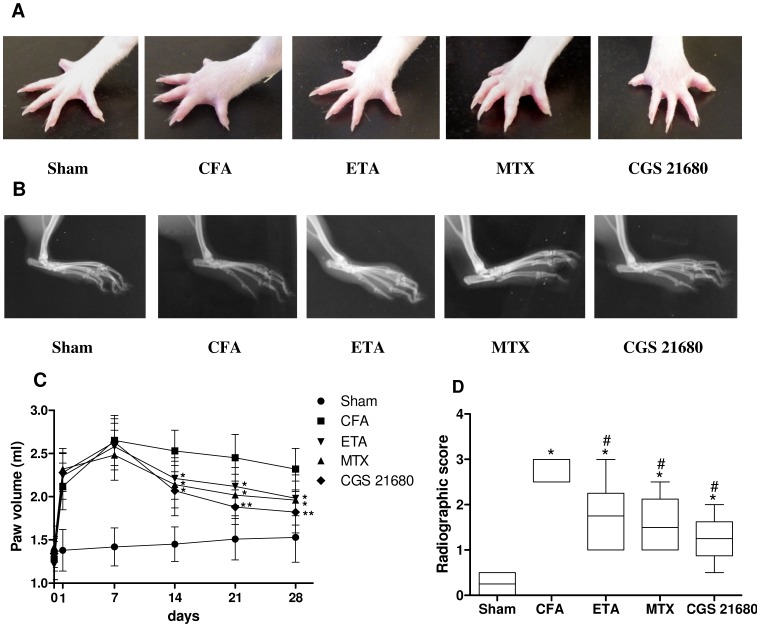
A significant decrease of paw swelling and radiographic damage in CFA-injected rats treated with CGS 21680 was detected. Representative photographs (A) and radiographs (B) of left hind paw from sham rats and CFA-injected rats in the absence or in the presence of chronic treatment with etanercept (ETA), methotrexate (MTX) or CGS 21680 at 28 days after CFA-injection. (C) Paw volume measurements of sham rats and CFA-injected rats untreated or treated with ETA, MTX or CGS 21680 at 0, 1, 7, 14, 21 and 28 days after CFA-injection. The results are presented as mean ± SD (n = 6 for each group). *, *p*<0.05 and **, *p*<0.01 vs CFA injected rats. (D) Radiographic score (grade 0–3) of the examined rats at 28 days after CFA-injection. The results are presented as median and interquartile range. *, *p*<0.01 vs sham rats; #, *p*<0.01 vs CFA-injected rats.

Fig. 3A shows representative image ofthe left hind paws of sham and CFA-injected rats on day 28. The injection of CFA evoked a marked paw swelling that peaked within 7 days and was sustained for 28 days. The left hind paw volume of CFA-injected rats increased from 1.28±0.24 ml to 2.65±0.29 ml, when evaluated 7 days after CFA-injection (Fig. 3C). The treatment of CFA-injected rats with the A_2A_AR agonist CGS 21680 resulted in a statistically significant decrease of paw swelling 14 days after CFA injection compared with untreated arthritic rats. This effect was comparable to those obtained treating the rats with a classic drug as MTX or with a biologic drug as etanercept. Interestingly, after 21 days an higher inhibition of paw swelling was observed in A_2A_AR agonist-treated rats respect to the rats treated with MTX or etanercept, with paw volume being 40% less than that in untreated CFA-injected rats. This major effect of CGS 21680 was still evident 28 days after CFA injection, with a paw volume not statistically different from sham rats. Fig. 3B shows the radiographic images of the left hind paws on day 28. In particular, CFA-injected rats developed definite joint space narrowing of the intertarsal joints, diffuse soft tissue swelling that included the digits, marked periosteal thickening, cystic enlargement of bone, and extensive erosions. The animal treated with MTX, etanercept or CGS 21680 exhibited a marked decrease of radiographic scores in comparison with untreated rats (Fig. 3D). Interestingly, rats treated with CGS 21680 showed less radiological osteoporosis and less destructive changes than those treated with MTX or etanercept. In addition, the animals that were treated with CGS 21680 maintained good health condition and were not significantly different in body weight from the rats of the sham group (data not shown).

### Ultrasonographic Assessment of Arthritis Progression in CFA-injected Rats

**Figure 4:**
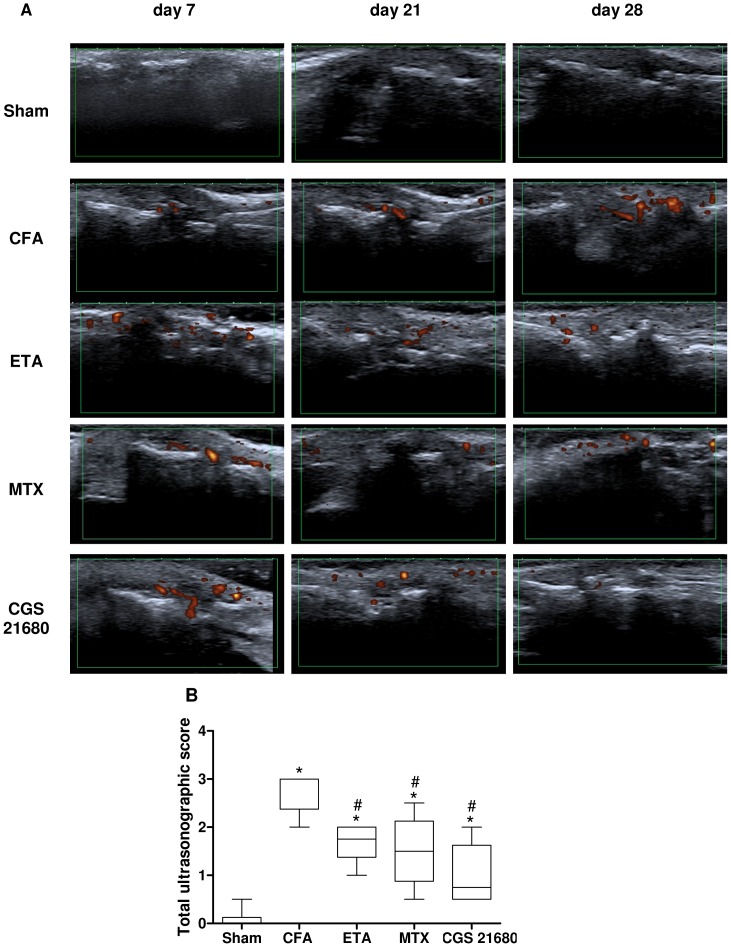
A reduction of the synovitis was present in CFA-injected rats after chronic treatment with CGS 21680. (A) Representative ultrasonographic (US) pictures of left hind paw andlongitudinal lateral view from sham rats and CFA-injected rats in the absence or in the presence of chronic treatment with etanercept (ETA), methotrexate (MTX) or CGS 21680 at 7, 21 and 28 days after CFA-injection. Total US score of the examined rats 28 days after CFA-injection. (B) Grey-scale and power doppler signals were assigned semiquantitatively and summarized in a total US score (grade 0–3). The results are presented as median and interquartile range (n = 6 for each group). *, *p*<0.01 vs sham rats; #, *p*<0.01 vs CFA-injected rats.

The US assessment showed the persistence of effusion and active synovitis in the CFA-injected rats at all the time points examined in comparison with sham rats. Notably, the animals treated with MTX, etanerceptor CGS 21680 exhibited a gradual reduction of the synovitis (Fig. 4A). The total US score decreased over the study period in rats treated with MTX or etanercept reaching a median value of 1.50 [0.88–2.13] or 1.75 [1.38–2.00] in the evaluation performed 28 days after CFA injection, respectively (Fig. 4B). At the same time point, CGS 21680-treated rats had an overall median score of 0.75 [0.50–1.63], showing a significant reduction as compared with untreated CFA-injected rats (overall US median score: 3.00 [2.38–3.00]) (Fig. 4B). In addition, rats treated with CGS 21680 showed low level US activity (power Doppler, PD ≤1) while rats treated with MTX or etanercept exhibited higher signs of destructive changes and erosion in comparison with rats treated with CGS 21680.

### Pain Responses in CFA-injected Rats After Different Pharmacological Treatments

**Figure 5:**
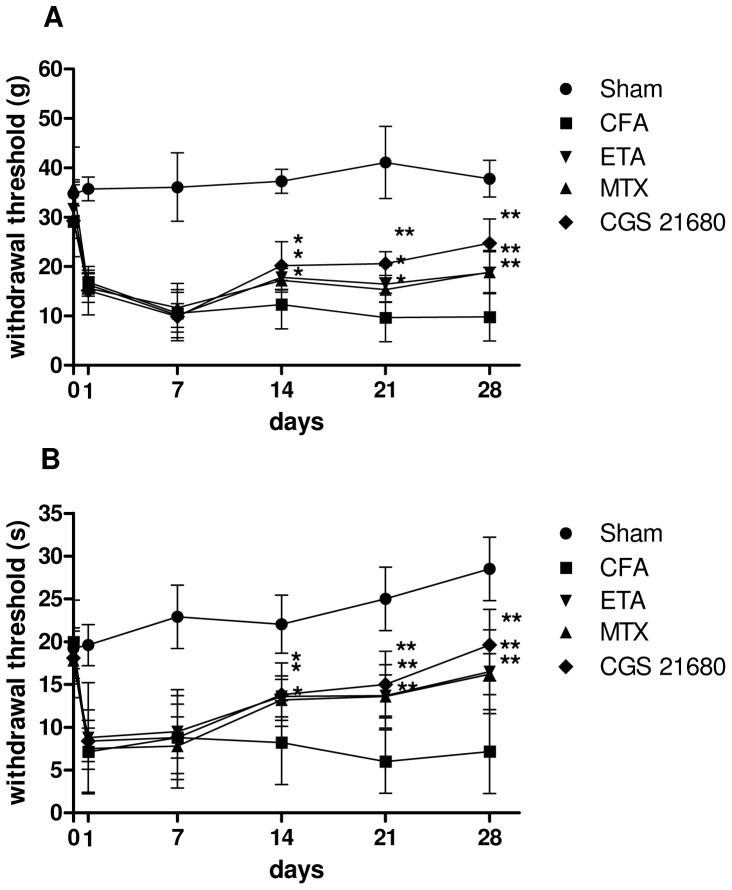
The treatment with CGS 21680 decreased the mechanical allodynia and thermal hyperalgesia in CFA-injected rats. Paw withdrawal threshold in mechanical allodynia (A) and in thermal hyperalgesia (B) in sham rats and CFA-injected rats in the absence or in the presence of chronic treatment with etanercept (ETA), methotrexate (MTX) or CGS 21680 at 0, 1, 7, 14, 21 and 28 days after CFA-injection. The results are presented as mean ± SD (n = 6 for each group). *, *p*<0.05 and **, *p*<0.01 vs CFA-injected rats.

The CFA-induced arthritic rat model is an extensively used laboratory model for the study of arthritic pain since the animals exhibit mechanical allodynia, thermal hyperalgesia and pain on joint movement which are the prominent features of arthritic pain in man [38]. In the present study, CFA-injected rats showed a significant reduction in paw withdrawal threshold compared to sham rats from 1 to 28 days after CFA injection (Fig. 5). In mechanical allodynia, the rats treated with MTX or etanercept (2 mg/kg or 5 mg/kg, respectively) increased the withdrawal threshold compared to CFA-injected rats from 14 to 28 days after arthritis induction. CGS 21680 treatment significantly increased (*p*<0.01) withdrawal threshold respect to CFA-injected rats showing a higher effect than MTX or etanercept on day 21 (Fig. 5A). Similarly, the treatment with MTX, etanercept or CGS 21680 decreased thermal hyperalgesia as compared to CFA control group (Fig. 5B). Interestingly, the anti-allodynic and anti-hyperalgesic effects of CGS 21680 confirmed the involvement of A_2A_AR stimulation in the reduction of arthritis-associated pain.

### A_2A_AR Expression in Lymphocytes and IL-10 Release in Serum from CFA-injected Rats

**Figure 6:**
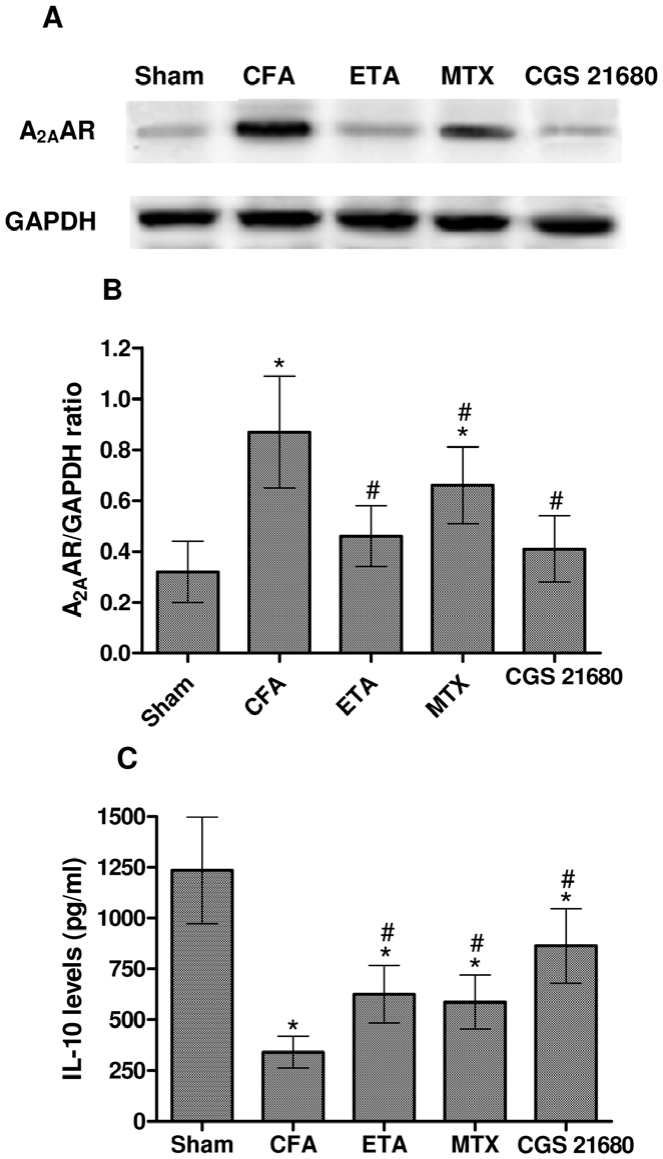
A_2A_ARs were up-regulated in lymphocytes from CFA-injected rats and were able to modulate IL-10 levels. Western blotting (A) and densitometric analysis (B) of A_2A_ARs in CFA-injected rats untreated or treated with etanercept (ETA), methotrexate (MTX) or CGS 21680 in comparison with sham rats. (C) ETA, MTX or CGS 21680 chronic treatment elicited an increase of IL-10 levels in serum of CFA-injected rats.The results are presented as mean ± SD (n = 6 for each group).*, *p*<0.01 vs sham rats; #, *p*<0.01 vs CFA-injected rats.

In lymphocytes from CFA-injected rats, Western blotting (Fig. 6A) and densitometric analysis (Fig. 6B) performed 28 days after CFA injection indicated a significant increase in A_2A_AR expression in comparison with sham rats. The treatment with MTX produced a significant reduction of A_2A_AR up-regulation although minor than those obtained after etanercept or CGS 21680 treatment. Moreover, IL-10 levels were measured in serum samples from sham and CFA-injected rats as reported in Fig. 6C. Reduced levels of IL-10 in CFA control group respect to sham rats were observed. In CFA-injected rats chronic treatment with CGS 21680 was able to restore IL-10 levels whereas MTX or etanercept had a weaker effect.

## Discussion

To date no clinical trials are present evaluating the effects of A_2A_AR agonists in RA patients since more preclinical studies are needed to better elucidate their pharmacological properties. RA treatment has progressed for the advent of biologic DMARDs even if the risks of infection, infusion-associated reactions or malignancy together to the high costs of these treatments could limit their clinical use [5].

In the present study, we have investigated the involvement of A_2A_ARs in RA by evaluating their affinity and density in lymphocytes from RA patients at different time points after the treatment with classic or biologic DMARDs. We have found an up-regulation of A_2A_ARs in untreated RA patients that was gradually reduced in function of the treatment time and in different ways depending on the type of drug used. These results complete our previous evidence regarding A_2A_ and A_3_ARs in blood cells from early RA and RA patients where an alteration of these receptors was found [31], [32] and highlight important information on the effect of biological treatments. The longitudinal study (from 0 to 24 months) performed for the first time in this paper aimed to assess the time-dependent changes of A_2A_AR expression in differentially treated RA patients. In MTX-treated RA patients A_2A_ARs were present in high levels at all the time points of treatment whilst in anti-TNFα-treated RA patients A_2A_AR density normalized to control values after 24 months of treatment. RA patients treated with RTX exhibited A_2A_AR binding characteristics similar to control values after 9 and until 24 months of treatment. It is tempting to speculate that the different behavior of examined drugs in RA patients on A_2A_AR density could be correlated with the several mechanisms of action involving the inflammatory process regulated by these heterogeneous therapies. DAS 28 values progressively reduced in function of the time of treatment and in different way respect to the type of drugs used with a significant reduction after 3 months of RTX treatment. Moreover, the time-dependent relationship between A_2A_ARs and DAS 28 in lymphocytes from RTX treated RA patients strongly suggested the involvement of A_2A_ARs in RA progression. Interestingly, high levels of DAS 28 indicating the presence of a marked inflammatory status are accompanied by an increased A_2A_AR density. These data suggest that A_2A_AR expression is sensibly affected by inflammation and tend to normalize with the disease remission. Thus, A_2A_ARs could be an useful tool to monitor RA progression following conventional or biologic therapies. On the other hand, A_2A_AR upregulation could be interpreted as a possible compensatory mechanism that is needed to opposite the inflammatory status. For this reason we have investigated the effect of an A_2A_AR agonist CGS 21680 on the production of the anti-inflammatory cytokine IL-10 in lymphocytes from RA patients. IL-10, a major immunoregulatory cytokine, is mainly produced by lymphocytes or macrophages and plays an important role in RA pathogenesis mediating the down-regulation of the inflammatory response. In particular, it has been demonstrated that IL-10 suppresses joint swelling and deformation as well as necrosis of cartilage in RA animal models [46]. We have found that CGS 21680 mediates a significant increase in IL-10 levels in cultured lymphocytes from RA patients confirming the link between the use of A_2A_AR agonist and the reduction of the inflammation. Moreover, CGS 21680 did not reduce cell viability and was not able to exert cytotoxic effects in human lymphocytes.

Based on these relevant results we have investigated the effect of A_2A_AR stimulation in a model of experimental arthritis represented by CFA-induced arthritic rats. Interestingly, CGS 21680 treatment was able to reduce the severity of arthritis being at least as efficacious as MTX or etanercept. In particular, CGS 21680 strongly decreased the clinical signs of arthritis induced by CFA injection in rats as demonstrated by radiological and US evaluation. It is well reported that the latter technique is more accurate than standard clinical examination at detecting synovitis in human RA [47], [48]. In this study we have applied for the first time, to our knowledge, the US evaluation in the adjuvant-induced arthritis model showing a marked reduction of total US score in rats treated with CGS 21680. The data from US assessment are in agreement with radiographic analysis suggesting the relevance of these technical approaches for the examination of arthritis damage in animal models.

It is well reported that enhanced pain perception is common among patients with RA [49]. The identification of novel pharmacological strategy that reduce the severity of the disease, could contribute to decrease the associated pain, improving general clinical and functional outcomes. In CFA-induced arthritis, A_2A_AR stimulation was able to decrease arthritis-associated pain as observed by the threshold increase in mechanical allodynia and in thermal hyperalgesia. These results demonstrated the effectiveness of CGS 21680 in adjuvant-induced model of arthritis in rats and are in agreement with those found using different experimental approaches in collagen-induced arthritis (CIA) in mice [25]. Recently, a phosphorylated A_2A_AR agonist was demonstrated to be a potent immunosuppressant in a model of arthritis acting by an up-regulation of CD73 and A_2A_AR expression [50].

To complete our study, we have verified in CFA-injected rats an A_2A_AR up-regulation similar to that found in RA patients confirming the relevance of this animal model to study the involvement of A_2A_ARs in the disease. Moreover, the anti-inflammatory effect of CGS 21680 was also demonstrated by the increase of IL-10 levels in serum from CFA-injected rats reflecting the data reported in lymphocytes from RA patients. This experimental evidence confirmed previous results obtained in human chondrocytes from RA patients where the stimulation of A_2A_ARs increased IL-10 release [24]. In addition, in a murine calvaria model of wear particle induced bone resorption, the treatment with CGS 21680 mediated a decrease of pro-inflammatory cytokines secretion, whereas IL-10 was markedly increased in bone [51]. These *in vivo* evidence suggested that the beneficial effect of CGS 21680 in experimental arthritis could be due, at least in part, to the increase of IL-10 which counterbalance the overexpression of inflammatory mediators. It is worth noting that the reduction of the inflammation could underlie the normalization of A_2A_ARs in lymphocytes from arthritic rats after CGS 21680 chronic treatment.

In conclusion, we have demonstrated the involvement of A_2A_ARs in RA pathogenesis based on the modulation of their expression in function of the time after different pharmacological treatments. Moreover, our *in vitro* and *in vivo* results highlight the role of A_2A_AR signalling as a protective anti-inflammatory system making A_2A_AR agonists an ideal and more physiological-like therapeutic alternative for the treatment of chronic autoimmune joint diseases such as RA. As a matter of fact, A_2A_AR agonists, mimicking an endogenous protective system, could have less limitation and side effects than DMARDs or biologic drugs although with a comparable effectiveness. These experimental results support the role of A_2A_AR as therapeutic target and strongly suggest the potential use of novel pharmacological approaches based on A_2A_AR stimulation in RA treatment.
